# Supplementary material for: Plasma proteomic signatures of social isolation and loneliness associated with morbidity and mortality
Source: Nat Hum Behav. 2025 Jan 3;9(3):569–83. doi: 10.1038/s41562-024-02078-1 (PMC11936835; doi:10.1038/s41562-024-02078-1)
Supplement: Supplementary file 1 — Supplementary Figs. 1–19. [file 41562_2024_2078_MOESM1_ESM.pdf]

nature human behaviour

Supplementary information https://doi.org/10.1038/s41562-024-02078-1

# Plasma proteomic signatures of social isolation and loneliness associated with morbidity and mortality

In the format provided by the authors and unedited

## Table of contents

Supplementary Fig. 1 Flow chart of participant selection. 2
Supplementary Fig. 2 PWAS of social isolation and loneliness in simple models. 3
Supplementary Fig. 3 Relationship between proteomic profiles of social isolation and loneliness. 4
Supplementary Fig. 4 Proteins nonlinearly associated with social isolation. 5
Supplementary Fig. 5 PWAS of social isolation and loneliness using ordered logistic regression. 6
Supplementary Fig. 6 PWAS of social isolation and loneliness in the randomly selected subset. 7
Supplementary Fig. 7 PWAS of social isolation and loneliness in Caucasians. 8
Supplementary Fig. 8 PWAS of social isolation and loneliness stratified by sex. 9
Supplementary Fig. 9 PWAS of social isolation and loneliness stratified by age. 11
Supplementary Fig. 10 PWAS of social isolation and loneliness accounting for depressive symptoms. 13
Supplementary Fig. 11 PWAS of social isolation and loneliness accounting for physical activity. 15
Supplementary Fig. 12 PWAS incorporating both social isolation and loneliness. 16
Supplementary Fig. 13 PWAS of four-group classification of social isolation and loneliness. 17
Supplementary Fig. 14 Results of cross-validation. 18
Supplementary Fig. 15 Results of protein co-expression network construction. 19
Supplementary Fig. 16 Comparison between protein co-expression networks constructed using different parameters. 20
Supplementary Fig. 17 Association of protein co-expression network, constructed by soft power of β=3, with social isolation and loneliness (N = 35,475). 21
Supplementary Fig. 18 Association of protein co-expression network, constructed by soft power of β=null, with social isolation and loneliness (N = 35,475). 22
Supplementary Fig. 19 Results of leave-one-out sensitivity analysis for MR using IVW method. 23

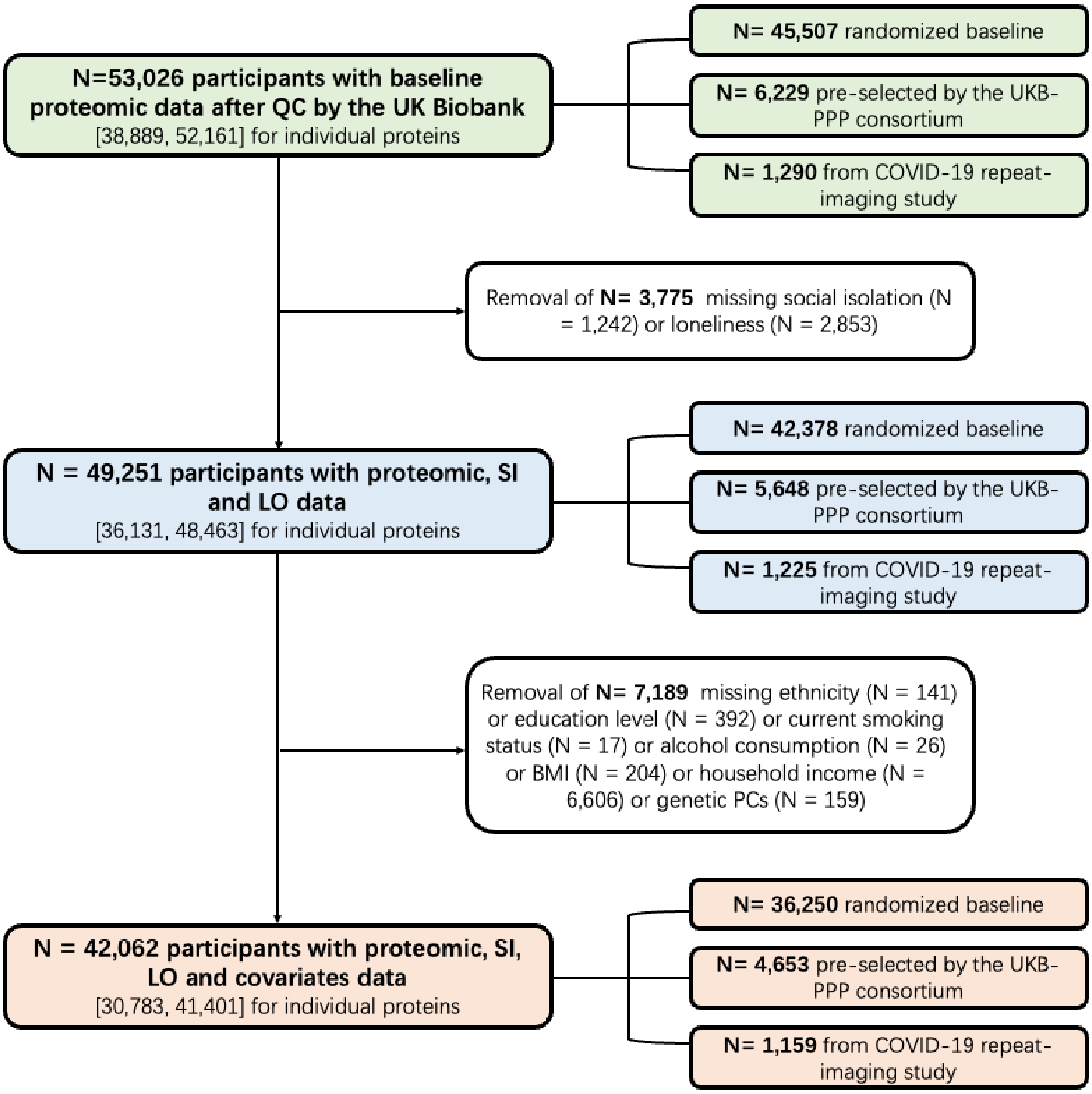


**Supplementary Fig. 1 Flow chart of participant selection.** UKB-PPP Consortium pre-selected participants were based on the Data-Field 30903. Participants from the COVID-19 repeat-imaging study were based on the Data-Field 41000.

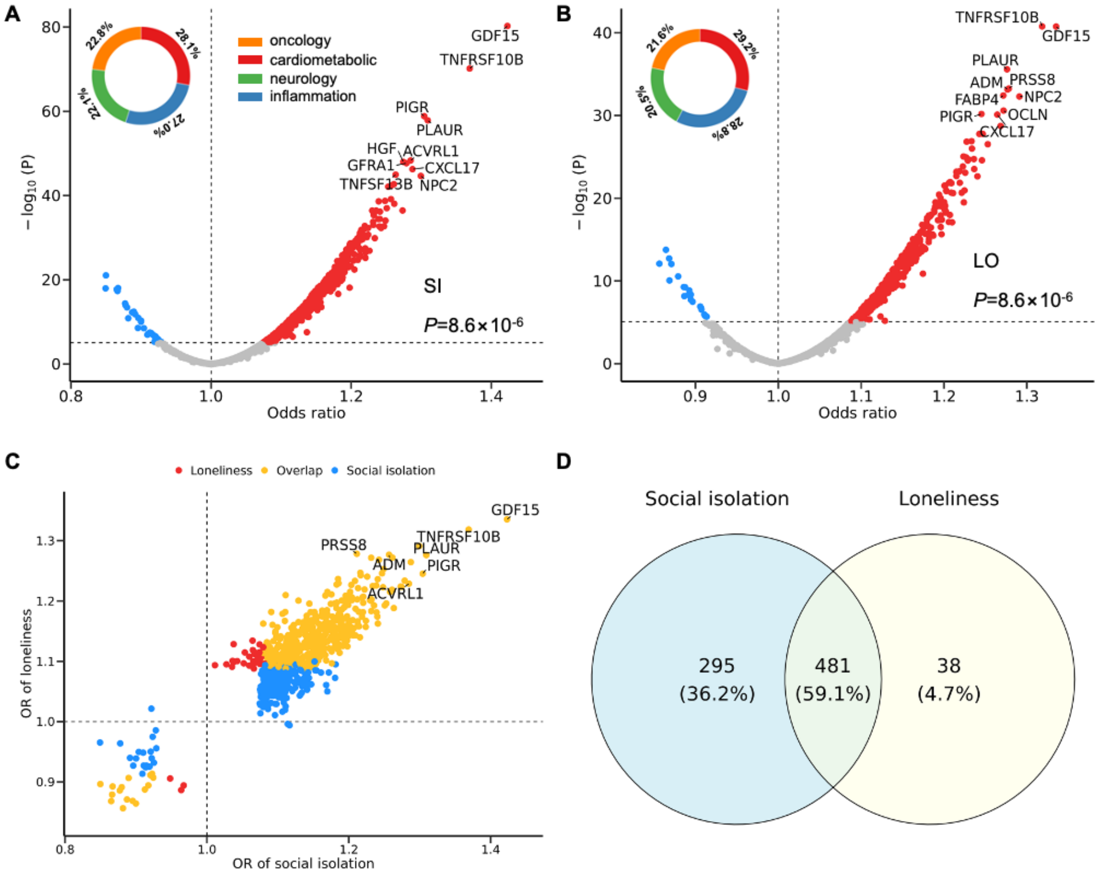


**Supplementary Fig. 2 PWAS of social isolation and loneliness in simple models.** The average sample size for the 2,920 proteins analyzed is 44,053, ranging from 36,026 to 48,304. Logistic regression models were adjusted for age, sex, site, batch, time gap between blood collection and protein measurement, and the first 20 genetic PCs. All statistical tests were two-sided. **A.** Volcano plot displaying the ORs (x axis) and -$\log_{10}$($P$-values) (y axis) for the association between protein abundance and social isolation. The pie chart depicts the proportions of identified proteins across four distinct panels. The dashed line represents Bonferroni correction ($P < 0.05/(2920 \times 2) = 8.6 \times 10^{-6}$). A total of 776 proteins were significant after Bonferroni correction. **B.** Volcano plot displaying the ORs (x axis) and -$\log_{10}$($P$-values) (y axis) for the association between protein abundance and loneliness. A total of 519 proteins were significant after Bonferroni correction. **C.** Scatter plot of ORs for proteins significantly associated with social isolation (x axis) and loneliness (y axis). **D.** Venn diagram showing the overlap of proteins significantly associated with social isolation and loneliness.

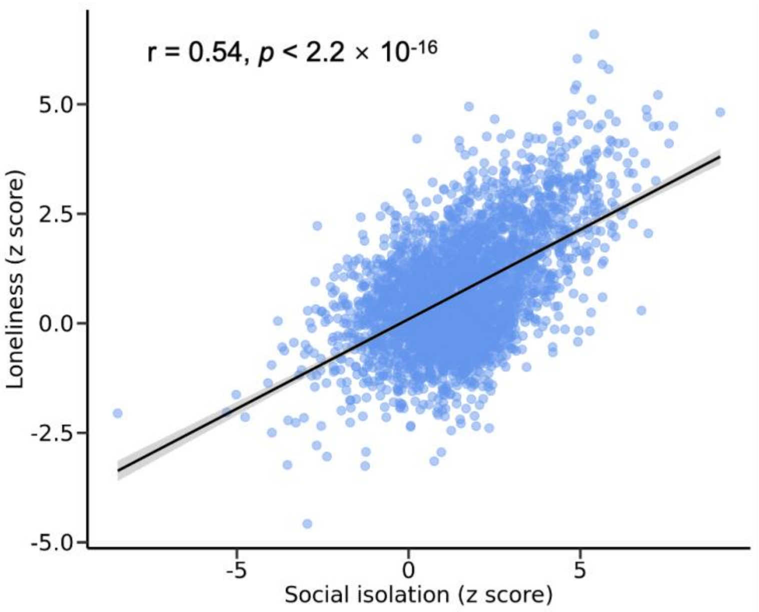


**Supplementary Fig. 3 Relationship between proteomic profiles of social isolation and loneliness.** The scatter plot depicts the relationship between the z-statistics for the association of proteins with social isolation (x axis) and loneliness (y axis). *Z* scores were derived from logistic regression models adjusted for age, sex, site, batch, time gap between blood collection and protein measurement, ethnicity, education level, household income, smoking, alcohol consumption, BMI, and the first 20 genetic PCs. The coefficient and *P*-value obtained from Person correlation analysis are shown. The shaded error band represents the 95% CIs.

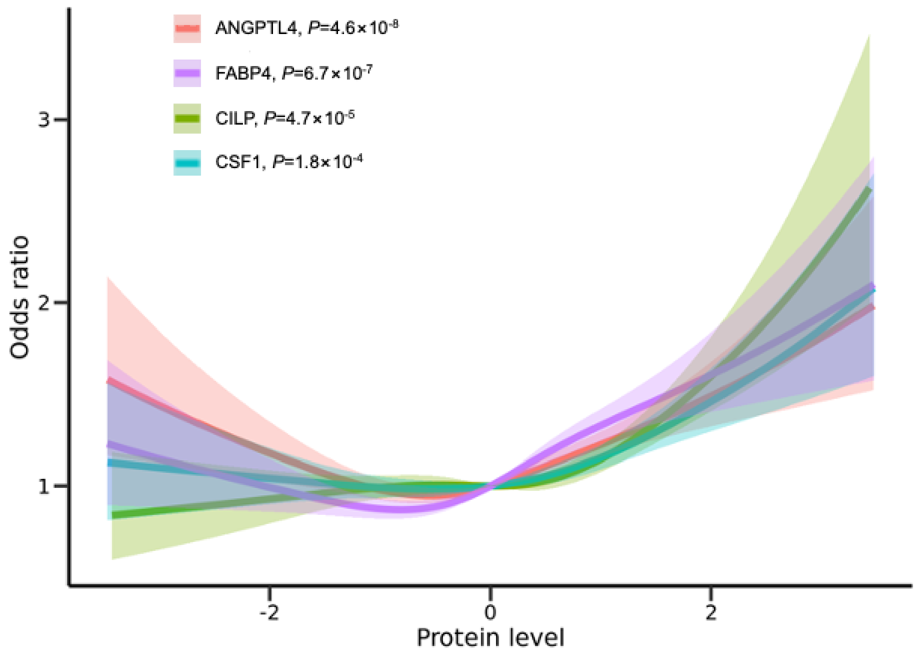


**Supplementary Fig. 4 Proteins nonlinearly associated with social isolation.** For proteins significantly associated with social isolation or loneliness identified by the PWAS, potential nonlinear relationships were assessed using multivariate restricted cubic splines regression. The models were adjusted for age, sex, site, batch, time gap between blood collection and protein measurement, ethnicity, education level, household income, smoking, alcohol consumption, BMI, and the first 20 genetic PCs. All statistical tests were two-sided. Nonlinear associations that remained significant after Bonferroni correction ($P < 0.05/(175+26) = 2.5\times10^{-4}$) are shown. The shaded error band represents the 95% CIs.

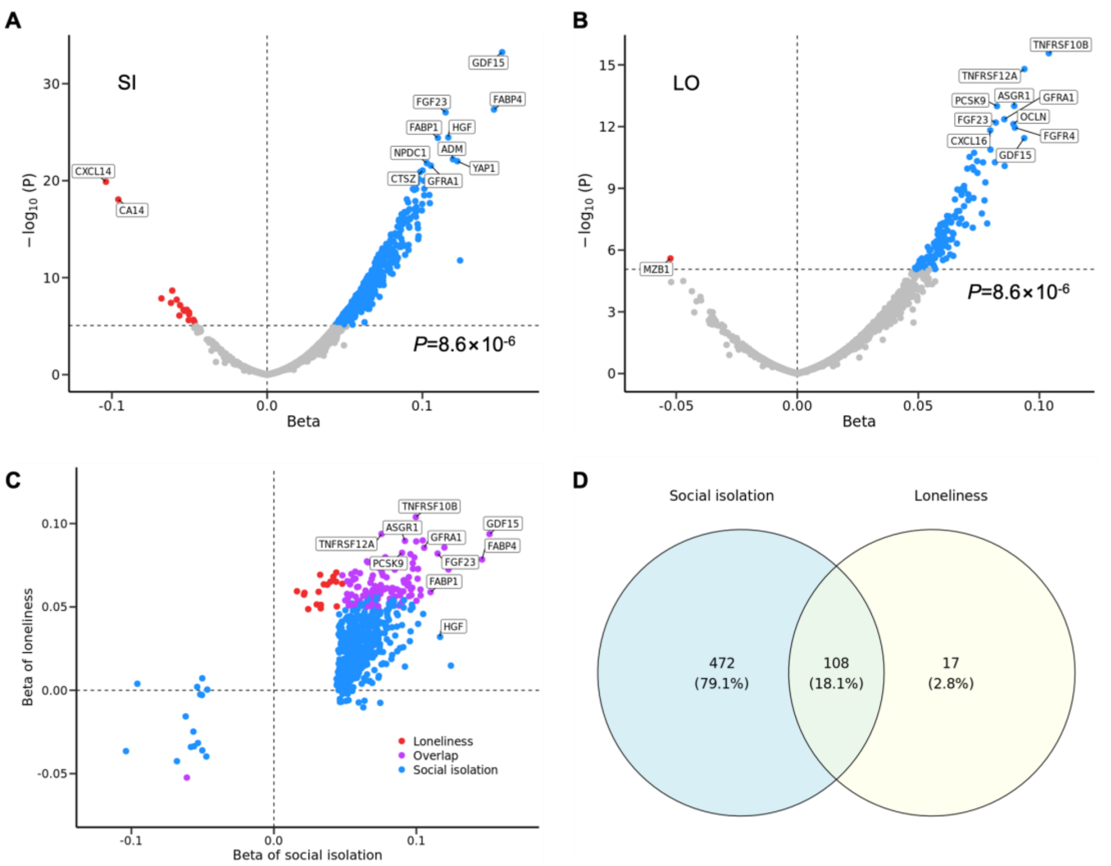


**Supplementary Fig. 5 PWAS of social isolation and loneliness using ordered logistic regression.** The average sample size for the 2,920 proteins analyzed is 37,704, ranging from 30,778 to 41,396. Social isolation scores ranged from 0 to 3, while loneliness scores ranged from 0 to 2. Covariates included age, sex, site, batch, time gap between blood collection and protein measurement, ethnicity, education level, household income, smoking, alcohol consumption, BMI, and the first 20 genetic PCs. All statistical tests were two-sided. **A.** Volcano plot displaying the Betas (x axis) and -$\log_{10}$($P$-values) (y axis) for the association between protein abundance and social isolation. The dashed line represents Bonferroni correction ($P < 0.05/(2920\times2) = 8.6\times10^{-6}$). A total of 580 proteins were significant after Bonferroni correction. 170 out of 175 proteins associated with social isolation identified by logistic regression remained significant. **B.** Volcano plot displaying the Betas (x axis) and -$\log_{10}$($P$-values) (y axis) for the association between protein abundance and loneliness. A total of 125 proteins were significant after Bonferroni correction. 23 out of 26 proteins associated with loneliness identified by logistic regression remained significant in the ordered logistic regression. **C.** Scatter plot of Betas for proteins significantly associated with social isolation (x axis) and loneliness (y axis). **D.** Venn diagram showing the overlap of proteins significantly associated with social isolation and loneliness.

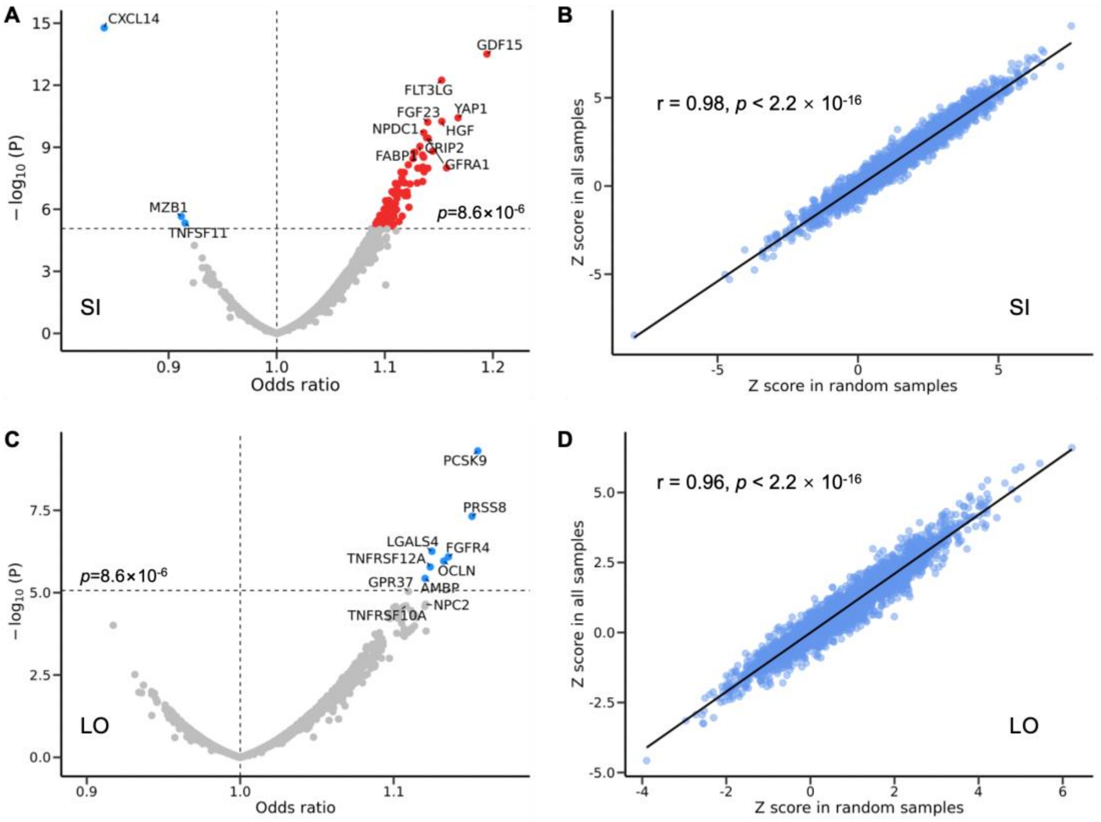


**Supplementary Fig. 6 PWAS of social isolation and loneliness in the randomly selected subset.** The average sample size for the 2,920 proteins analyzed is 33,003, ranging from 27,396 to 35,660. Logistic regression models were adjusted for age, sex, site, batch, time gap between blood collection and protein measurement, ethnicity, education level, household income, smoking, alcohol consumption, BMI, and the first 20 genetic PCs. All statistical tests were two-sided. **A.** Volcano plot displaying the ORs (x axis) and $-\log_{10}(P\text{-values})$ (y axis) for the association between protein abundance and social isolation. The dashed line represents Bonferroni correction ($P < 0.05/(2920\times2) = 8.6\times10^{-6}$). A total of 112 proteins were significant after Bonferroni correction. **B.** Scatter plot depicting the relationship between the z-statistics for the association of proteins with social isolation in random subset (x axis) and social isolation in all participants (y axis). The coefficient and *P*-value obtained from Person correlation analysis are shown. The shaded error band represents the 95% CIs. **C.** Volcano plot displaying the ORs (x axis) and $-\log_{10}(P\text{-values})$ (y axis) for the association between protein abundance and loneliness. Seven proteins were significant after Bonferroni correction. **D.** Scatter plot depicting the relationship between the z-statistics for the association of proteins with loneliness in random subset (x axis) and loneliness in all participants (y axis).

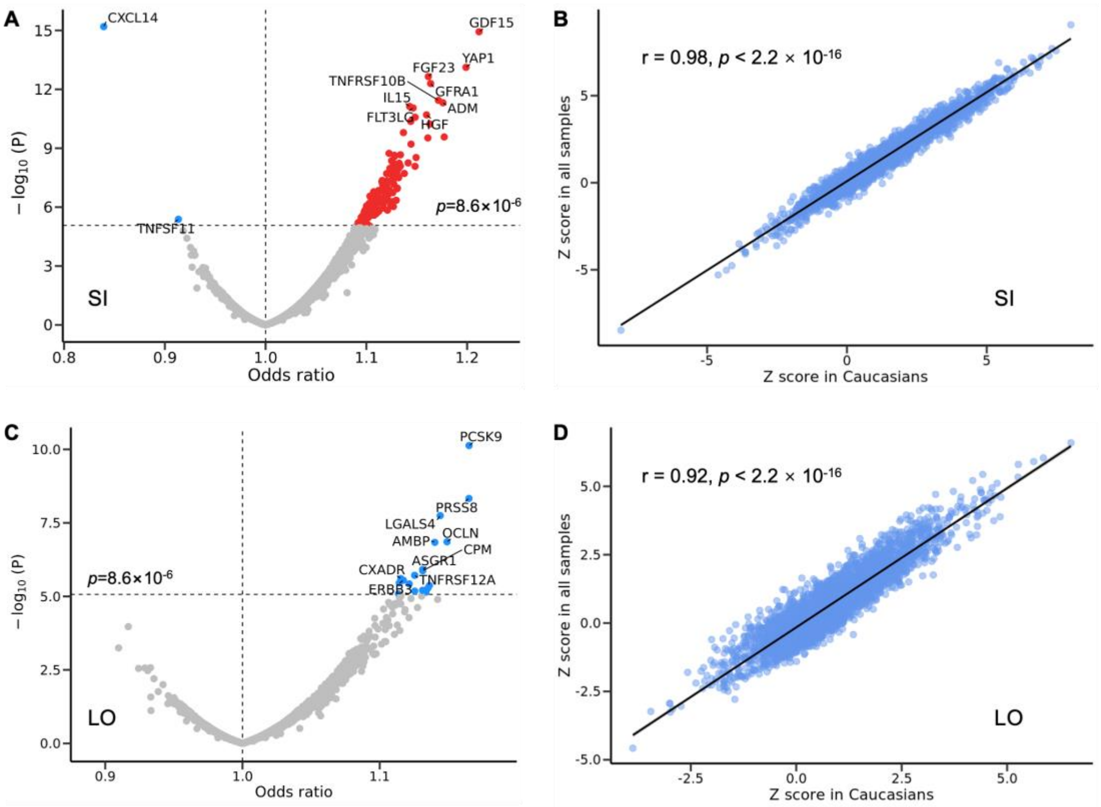


**Supplementary Fig. 7 PWAS of social isolation and loneliness in Caucasians.** Genetic ethnic grouping is based on the Data-Field 22006. The average sample size for the 2,920 proteins analyzed is 31,964, ranging from 26,037 to 35,140. Logistic regression models were adjusted for age, sex, site, batch, time gap between blood collection and protein measurement, education level, household income, smoking, alcohol consumption, BMI, and the first 20 genetic PCs. All statistical tests were two-sided. **A.** Volcano plot displaying the ORs (x axis) and -$\log_{10}$($P$-values) (y axis) for the association between protein abundance and social isolation. The dashed line represents Bonferroni correction ($P$ < 0.05/(2920×2) = 8.6×$10^{-6}$). A total of 153 proteins were significant after Bonferroni correction. 140 out of 175 significant proteins associated with social isolation identified in the full sample remained significant after Bonferroni correction, and all remained significant after FDR correction. **B.** Scatter plot depicting the relationship between the z-statistics for the association of proteins with social isolation in Caucasian subset (x axis) and social isolation in all participants (y axis). The coefficient and $P$-value obtained from Person correlation analysis are shown. The shaded error band represents the 95% CIs. **C.** Volcano plot displaying the ORs (x axis) and -$\log_{10}$($P$-values) (y axis) for the association between protein abundance and loneliness. Eighteen proteins were significant after Bonferroni correction. 14 out of 26 significant proteins associated with loneliness identified in the full sample remained significant after Bonferroni correction, and all remained significant after FDR correction. **D.** Scatter plot depicting the relationship between the z-statistics for the association of proteins with loneliness in Caucasian subset (x axis) and loneliness in all participants (y axis).

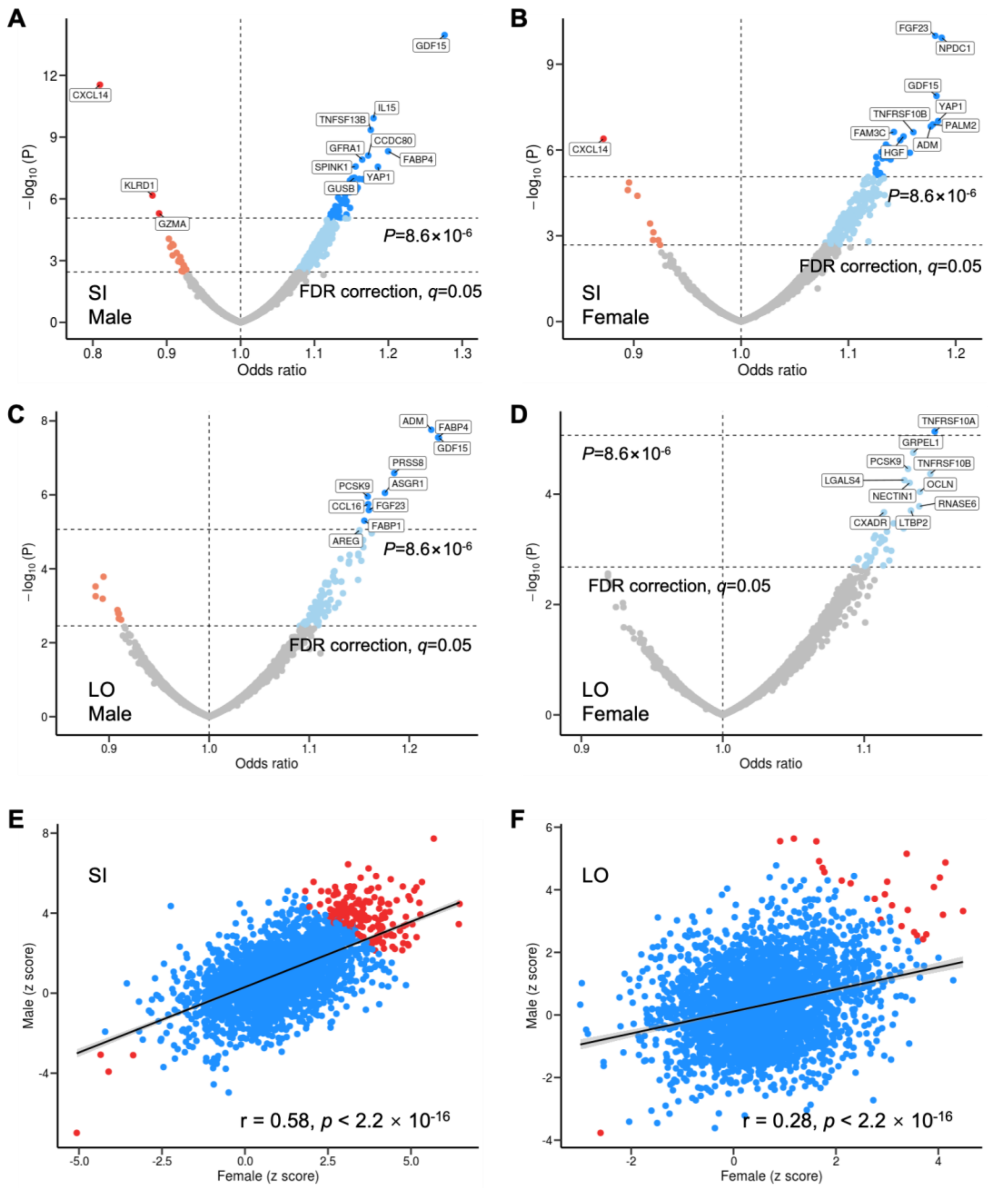


**Supplementary Fig. 8 PWAS of social isolation and loneliness stratified by sex.** The average sample size for the 2,920 proteins analyzed is 17,969, ranging from 14,700 to 19,751, in males, and 19,735, ranging from 16,078 to 21,645, in females. Logistic regression models were adjusted for age, site, batch, time gap between blood collection and protein measurement, ethnicity, education level, household income, smoking, alcohol consumption, BMI, and the first 20 genetic PCs. All statistical tests were two-sided. **A.** Volcano plot displaying the ORs (x axis) and -$\log_{10}$($P$-values) (y axis) for the association between protein abundance and social isolation in males. Dashed lines indicate the thresholds for Bonferroni ($P < 0.05/(2920 \times 2) = 8.6 \times 10^{-6}$) and FDR corrections ($q < 0.05$) when considering social isolation and loneliness simultaneously. **B.** Volcano plot displaying the ORs (x axis) and -$\log_{10}$($P$-values) (y axis) for the association between protein abundance and social isolation in females. **C.** Volcano plot displaying the ORs (x axis) and -$\log_{10}$($P$-values) (y axis) for the association between protein abundance and loneliness in males. **D.** Volcano plot displaying

the ORs (x axis) and -$\log_{10}$($P$-values) (y axis) for the association between protein abundance and loneliness in females. **E.** Scatter plot depicting the relationship between the z-statistics for the association of proteins with social isolation in females (x axis) and social isolation in males (y axis). The coefficient and $P$-value obtained from Person correlation analysis are shown. The shaded error band represents the 95% CIs. Red points indicate the 175 identified proteins significantly related to social isolation in the overall population. **F.** Scatter plot depicting the relationship between the z-statistics for the association of proteins with loneliness in females (x axis) and loneliness in males (y axis). Red points indicate the 26 identified proteins significantly related to loneliness in the overall population.

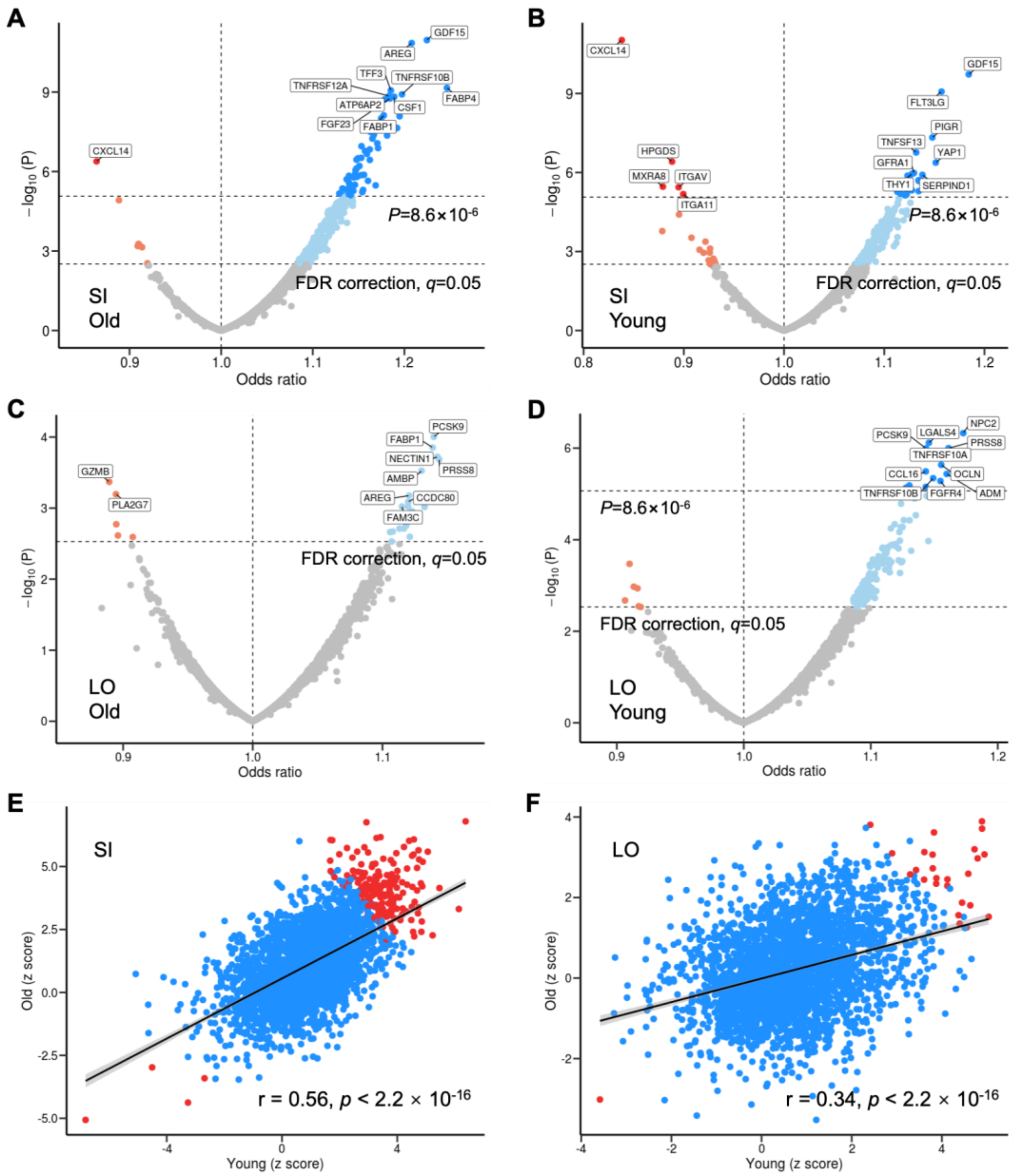


**Supplementary Fig. 9 PWAS of social isolation and loneliness stratified by age.** The average sample size for the 2,920 proteins analyzed is 16,054, ranging from 13,111 to 17,611, in the older group (⩾ 60 years), and 21,650, ranging from 17,667 to 23,785, in the younger group (< 60 years). Logistic regression models were adjusted for age, sex, site, batch, time gap between blood collection and protein measurement, ethnicity, education level, household income, smoking, alcohol consumption, BMI, and the first 20 genetic PCs. All statistical tests were two-sided. **A.** Volcano plot displaying the ORs (x axis) and -$\log_{10}$($P$-values) (y axis) for the association between protein abundance and social isolation in the older group. Dashed lines indicate the thresholds for Bonferroni ($P < 0.05/(2920 \times 2) = 8.6 \times 10^{-6}$) and FDR corrections ($q < 0.05$) when considering social isolation and loneliness simultaneously. **B.** Volcano plot displaying the ORs (x axis) and -$\log_{10}$($P$-values) (y axis) for the association between protein abundance and social isolation in the younger group. **C.** Volcano plot displaying the ORs (x axis) and -$\log_{10}$($P$-values) (y axis) for the association between protein abundance and loneliness in the older group. **D.** Volcano plot displaying the ORs (x axis) and -$\log_{10}$($P$-values) (y axis) for the association between protein abundance and loneliness

in the younger group. **E.** Scatter plot depicting the relationship between the z-statistics for the association of proteins with social isolation in the younger group (x axis) and social isolation in the older group (y axis). The coefficient and *P*-value obtained from Person correlation analysis are shown. The shaded error band represents the 95% CIs. Red points indicate the 175 identified proteins significantly related to social isolation in the overall population. **F.** Scatter plot depicting the relationship between the z-statistics for the association of proteins with loneliness in the younger group (x axis) and loneliness in the older group (y axis). Red points indicate the 26 identified proteins significantly related to loneliness in the overall population.

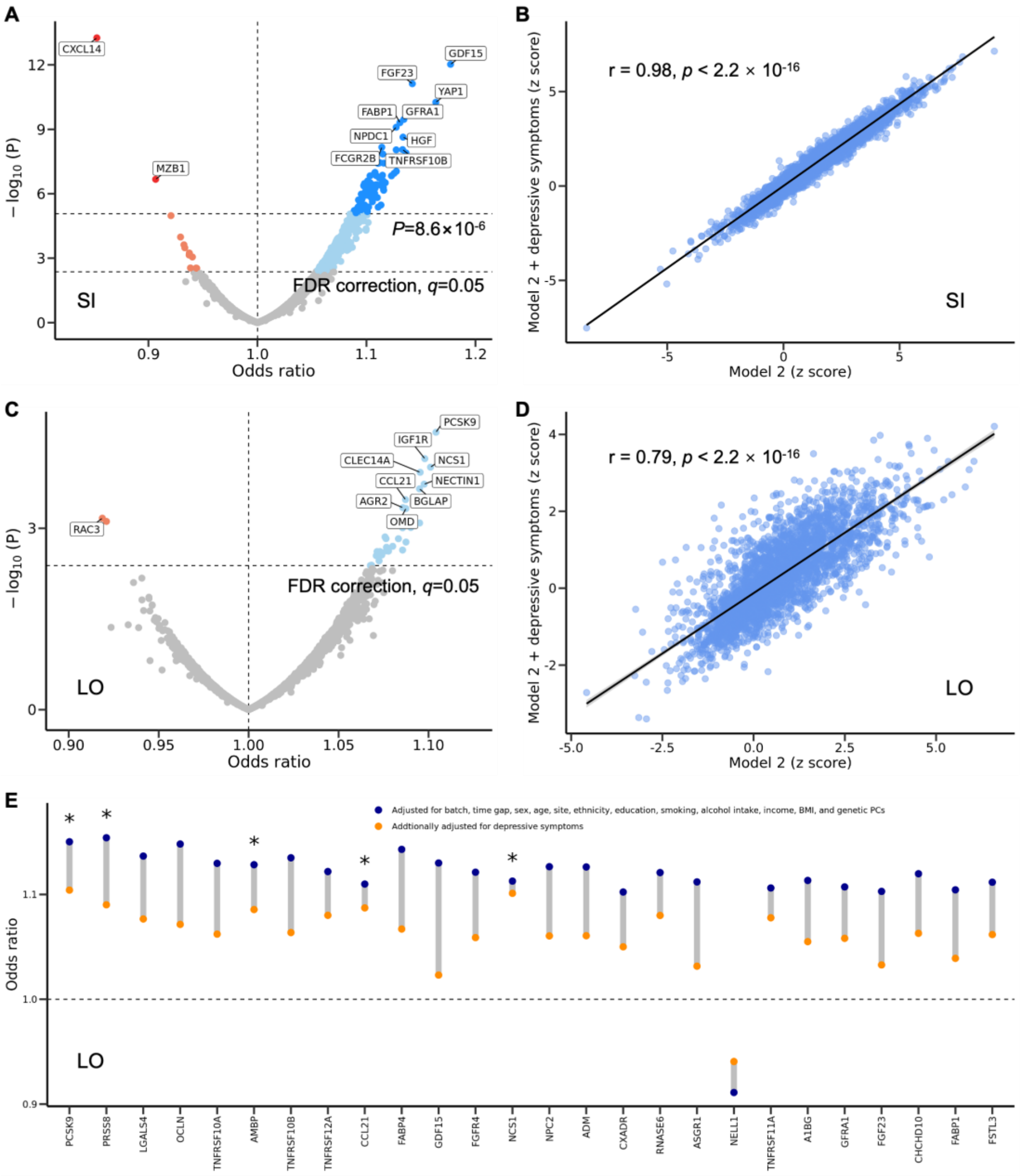


**Supplementary Fig. 10 PWAS of social isolation and loneliness accounting for depressive symptoms.** The average sample size for the 2,920 proteins analyzed is 34,755, ranging from 28,353 to 38,174. Logistic regression models were adjusted for age, sex, site, batch, time gap between blood collection and protein measurement, ethnicity, education level, household income, smoking, alcohol consumption, BMI, the first 20 genetic PCs, and depressive symptoms. All statistical tests were two-sided. **A.** Volcano plot displaying the ORs (x axis) and -$\log_{10}$($P$-values) (y axis) for the association between protein abundance and social isolation. Dashed lines indicate the thresholds for Bonferroni ($P < 0.05/(2920 \times 2) = 8.6 \times 10^{-6}$) and FDR corrections ($q < 0.05$) when considering social isolation and loneliness simultaneously. A total of 87 proteins were significant after Bonferroni correction and 471 proteins were significant after FDR correction. **B.** Scatter plot depicting the relationship between the z-statistics for the association of proteins with social isolation in the primary model (x axis) and social isolation further controlling for depressive symptoms (y axis). The coefficient and $P$-value obtained from Person correlation analysis are shown. The shaded

error band represents the 95% CIs. **C.** Volcano plot displaying the ORs (x axis) and -$\log_{10}$(*P*-values) (y axis) for the association between protein abundance and loneliness. No protein was significant after Bonferroni correction, but 31 proteins were significant after FDR correction. **D.** Scatter plot depicting the relationship between the z-statistics for the association of proteins with loneliness in the primary model (x axis) and loneliness further controlling for depressive symptoms (y axis). The shaded error band represents the 95% CIs. **E.** The alteration in ORs for the association between proteins and loneliness was assessed with or without adjustment for depressive symptoms. Proteins with a significance level of unadjusted $P < 0.001$ after additional adjustment for depression were marked by asterisks (PCSK9: $P = 2.6\times10^{-5}$; PRSS8: $P = 9.7\times10^{-4}$; AMBP: $P = 9.8\times10^{-4}$; CCL21: $P = 3.4\times10^{-4}$; NCS1: $P = 9.7\times10^{-5}$;).

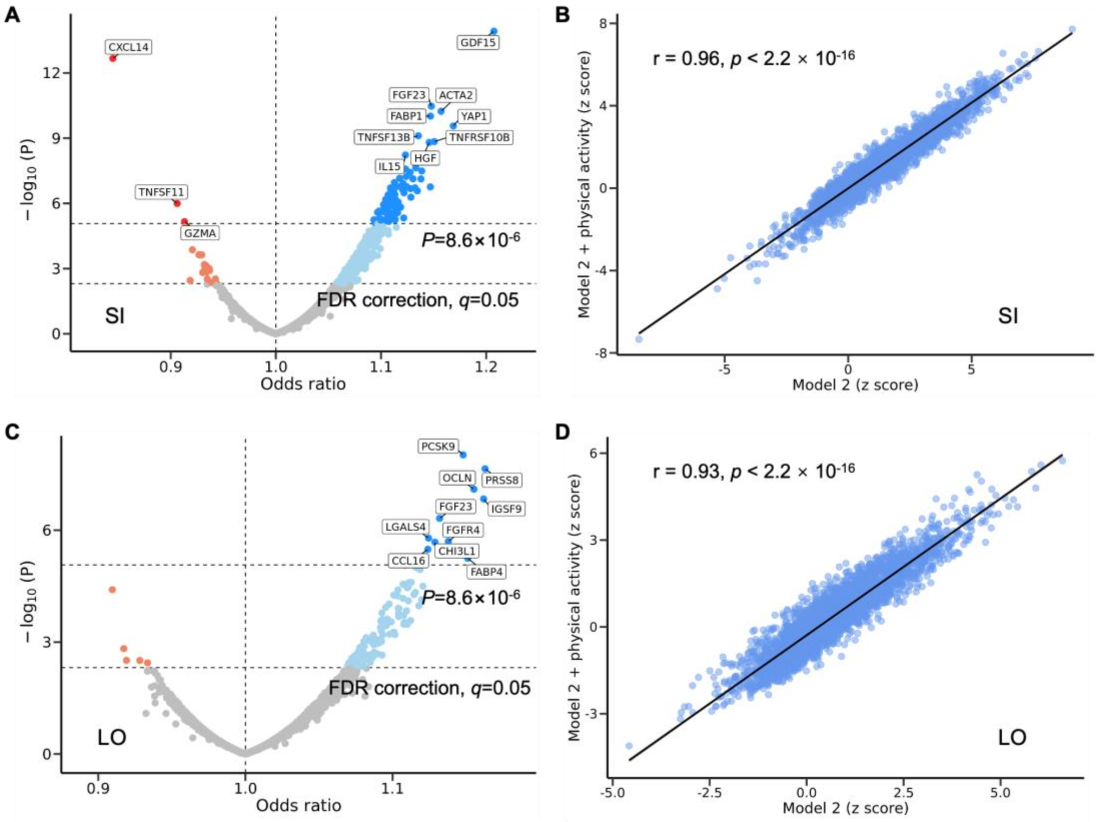


**Supplementary Fig. 11 PWAS of social isolation and loneliness accounting for physical activity.** The average sample size for the 2,920 proteins analyzed is 30,953, ranging from 25,257 to 33,991. Logistic regression models were adjusted for age, sex, site, batch, time gap between blood collection and protein measurement, ethnicity, education level, household income, smoking, alcohol consumption, BMI, the first 20 genetic PCs, and total MET minutes. All statistical tests were two-sided. **A.** Volcano plot displaying the ORs (x axis) and -log$_{10}$($P$-values) (y axis) for the association between protein abundance and social isolation. Dashed lines indicate the thresholds for Bonferroni ($P < 0.05/(2920×2) = 8.6×10^{-6}$) and FDR corrections ($q < 0.05$) when considering social isolation and loneliness simultaneously. A total of 92 proteins were significant after Bonferroni correction, and 436 proteins were significant after FDR correction. 88 out of 175 significant proteins associated with social isolation in the primary model remained significant after Bonferroni correction, and 173 remained significant after FDR correction. **B.** Scatter plot depicting the relationship between the z-statistics for the association of proteins with social isolation in the primary model (x axis) and social isolation further controlling for total MET minutes (y axis). The coefficient and $P$-value obtained from Person correlation analysis are shown. The shaded error band represents the 95% CIs. **C.** Volcano plot displaying the ORs (x axis) and -log$_{10}$($P$-values) (y axis) for the association between protein abundance and loneliness. A total of ten proteins were significant after Bonferroni correction, and 134 proteins were significant after FDR correction. 7 out of 26 significant proteins associated with loneliness in the primary model remained significant after Bonferroni correction, and all remained significant after FDR correction. **D.** Scatter plot depicting the relationship between the z-statistics for the association of proteins with loneliness in the primary model (x axis) and loneliness further controlling for total MET minutes (y axis).

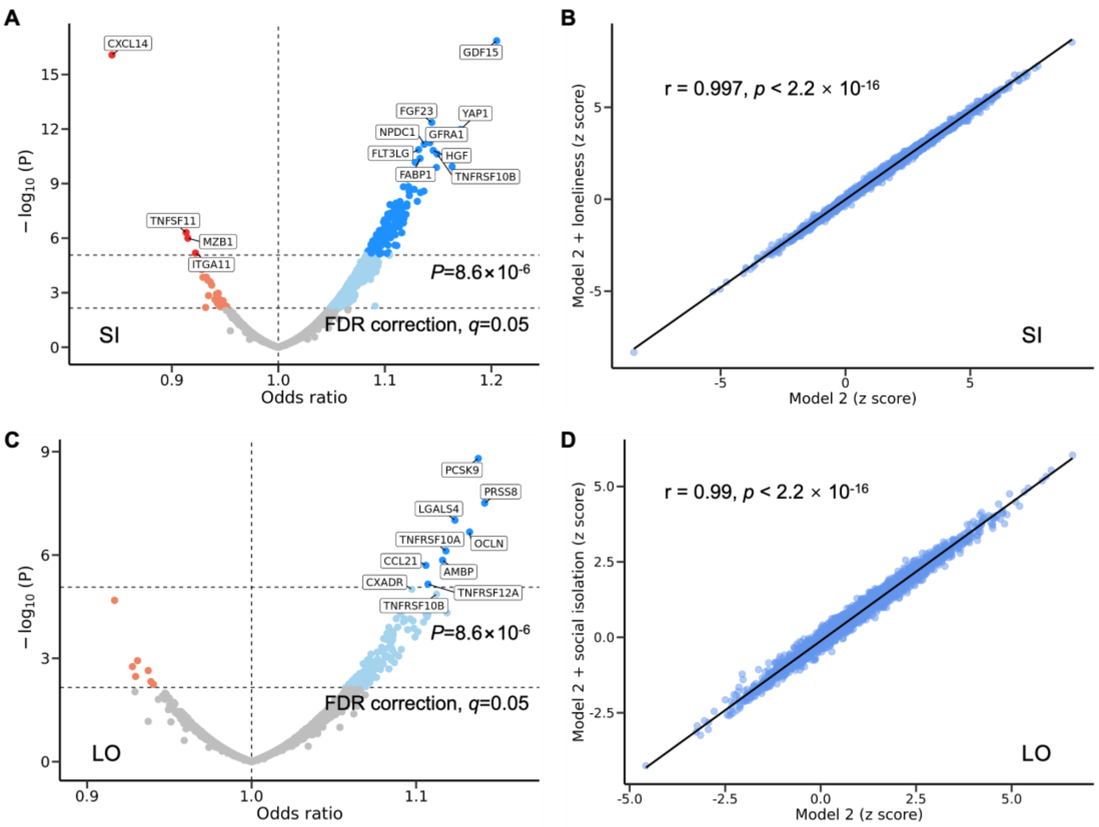


**Supplementary Fig. 12 PWAS incorporating both social isolation and loneliness.** The average sample size for the 2,920 proteins analyzed is 37,704, ranging from 30,778 to 41,396. Other covariates include age, sex, site, batch, time gap between blood collection and protein measurement, ethnicity, education level, household income, smoking, alcohol consumption, BMI, and the first 20 genetic PCs. All statistical tests were two-sided. **A.** Volcano plot displaying the ORs (x axis) and $-\log_{10}$($P$-values) (y axis) for the association between protein abundance and social isolation. Dashed lines indicate the thresholds for Bonferroni ($P < 0.05/(2920\times2) = 8.6\times10^{-6}$) and FDR corrections ($q < 0.05$) when considering social isolation and loneliness simultaneously. A total of 136 proteins were significant after Bonferroni correction and 655 proteins were significant after FDR correction. **B.** Scatter plot depicting the relationship between the z-statistics for the association of proteins with social isolation in the primary model (x axis) and social isolation further controlling for loneliness (y axis). The coefficient and $P$-value obtained from Person correlation analysis are shown. The shaded error band represents the 95% CIs. **C.** Volcano plot displaying the ORs (x axis) and $-\log_{10}$($P$-values) (y axis) for the association between protein abundance and loneliness. Eight proteins were significant after Bonferroni correction and 164 proteins were significant after FDR correction. **D.** Scatter plot depicting the relationship between the z-statistics for the association of proteins with loneliness in the primary model (x axis) and loneliness further controlling for social isolation (y axis).

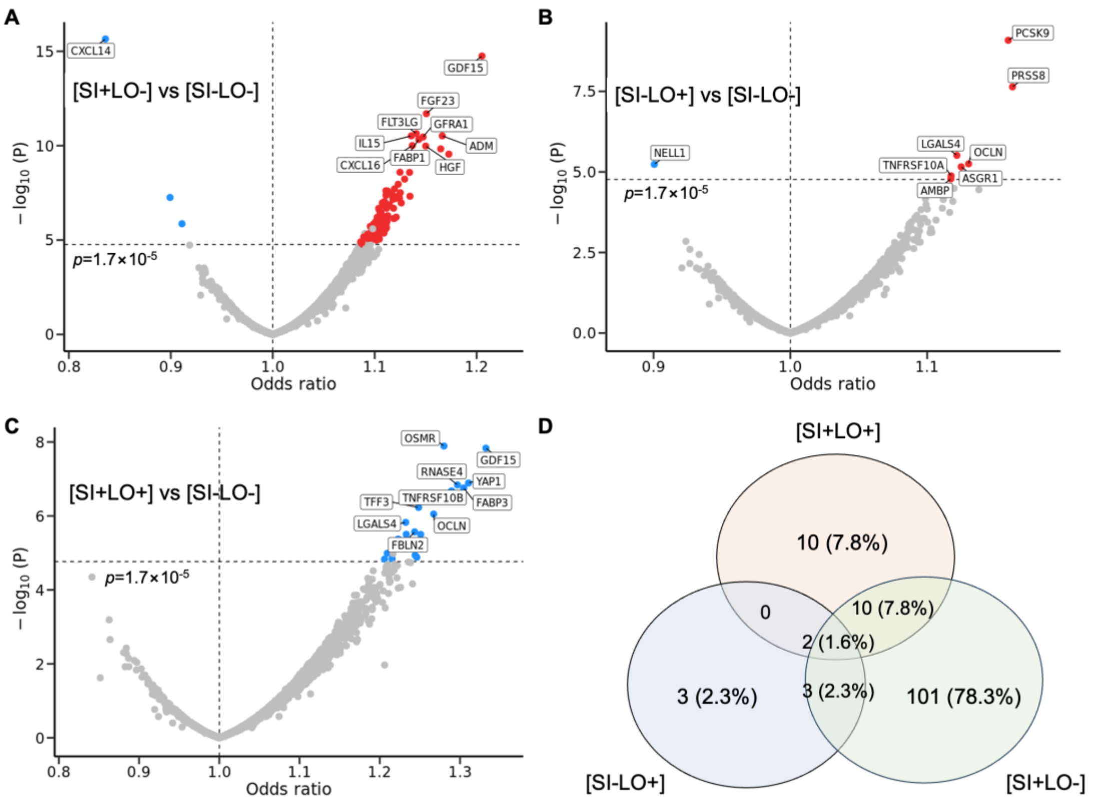


**Supplementary Fig. 13 PWAS of four-group classification of social isolation and loneliness.** The average sample size for the 2,920 proteins analyzed is 37,704, ranging from 30,778 to 41,396. Participants were categorized into four groups: neither isolated nor lonely [SI-LO-], socially isolated but not lonely [SI+LO-], not isolated but lonely [SI-LO+], and socially isolated and lonely [SI+LO+]. Multinominal logistic regression models were adjusted for age, sex, site, batch, time gap between blood collection and protein measurement, ethnicity, education level, household income, smoking, alcohol consumption, BMI, and the first 20 genetic PCs. The [SI-LO-] group served as the reference. All statistical tests were two-sided. A likelihood ratio test assessed the overall association of protein levels in comparison to the model with covariates only. Significant proteins associated with a specific group required both a significant model fit and a significant coefficient after Bonferroni correction ($P < 0.05/2920 = 1.7\times10^{-5}$). **A.** Volcano plot displaying the ORs (x axis) and -log$_{10}$($P$-values) (y axis) for proteins compared between [SI+LO-] and [SI-LO-]. The dashed line indicates the threshold for Bonferroni correction. A total of 116 proteins were significant after Bonferroni correction. **B.** Volcano plot displaying the ORs (x axis) and -log$_{10}$($P$-values) (y axis) for proteins compared between [SI-LO+] and [SI-LO-]. Eight proteins were significant after Bonferroni correction. **C.** Volcano plot displaying the ORs (x axis) and -log$_{10}$($P$-values) (y axis) for proteins compared between [SI+LO+] and [SI-LO-]. Twenty-two proteins were significant after Bonferroni correction. **D.** Venn diagram showing the overlap of significant proteins identified from the comparisons between the three groups and the reference.

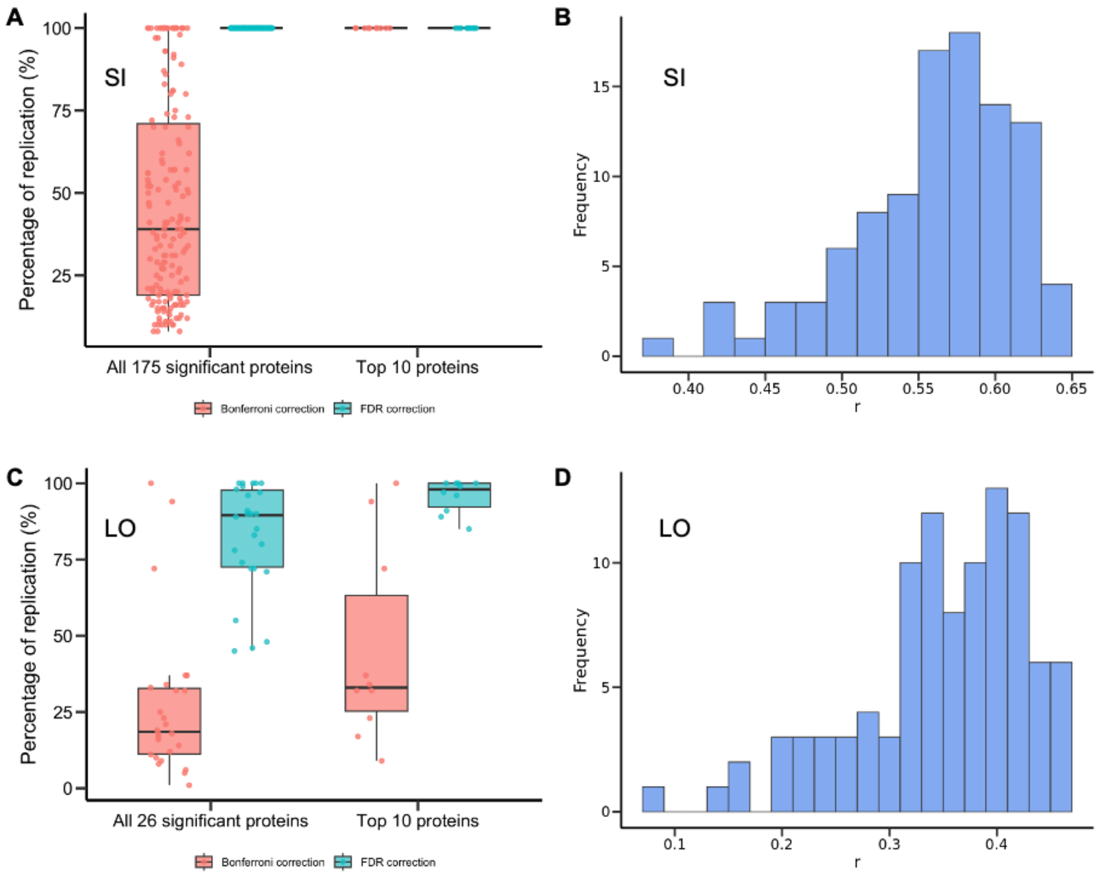


**Supplementary Fig. 14 Results of cross-validation.** Social isolation, loneliness, and control groups were randomly divided to ensure similar sample sizes for each group in the two split datasets. Full adjusted PWAS analyses were performed separately on the two datasets, and this process was repeated 100 times. **A.** Box plots showing the percentage of replication for all 175 proteins significantly related to social isolation and the top 10 proteins most strongly related to social isolation across 100 iterations. Each dot represents an individual protein. Red boxes indicate Bonferroni correction, and blue boxes indicate FDR correction. The midline of each box indicates the median of the data, the edges of the box represent the first and third quartiles, and the whiskers extend to the minimum and maximum values within 1.5 times the interquartile range from the quartiles. **B.** The distribution of the correlation coefficients for proteomic associative patterns related to social isolation identified in the two randomly split samples. **C.** Box plots showing the percentage of replication for all 26 proteins significantly related to loneliness and the top 10 proteins most strongly related to loneliness across 100 iterations. **D.** The distribution of the correlation coefficients for proteomic associative patterns related to loneliness identified in the two randomly split samples.

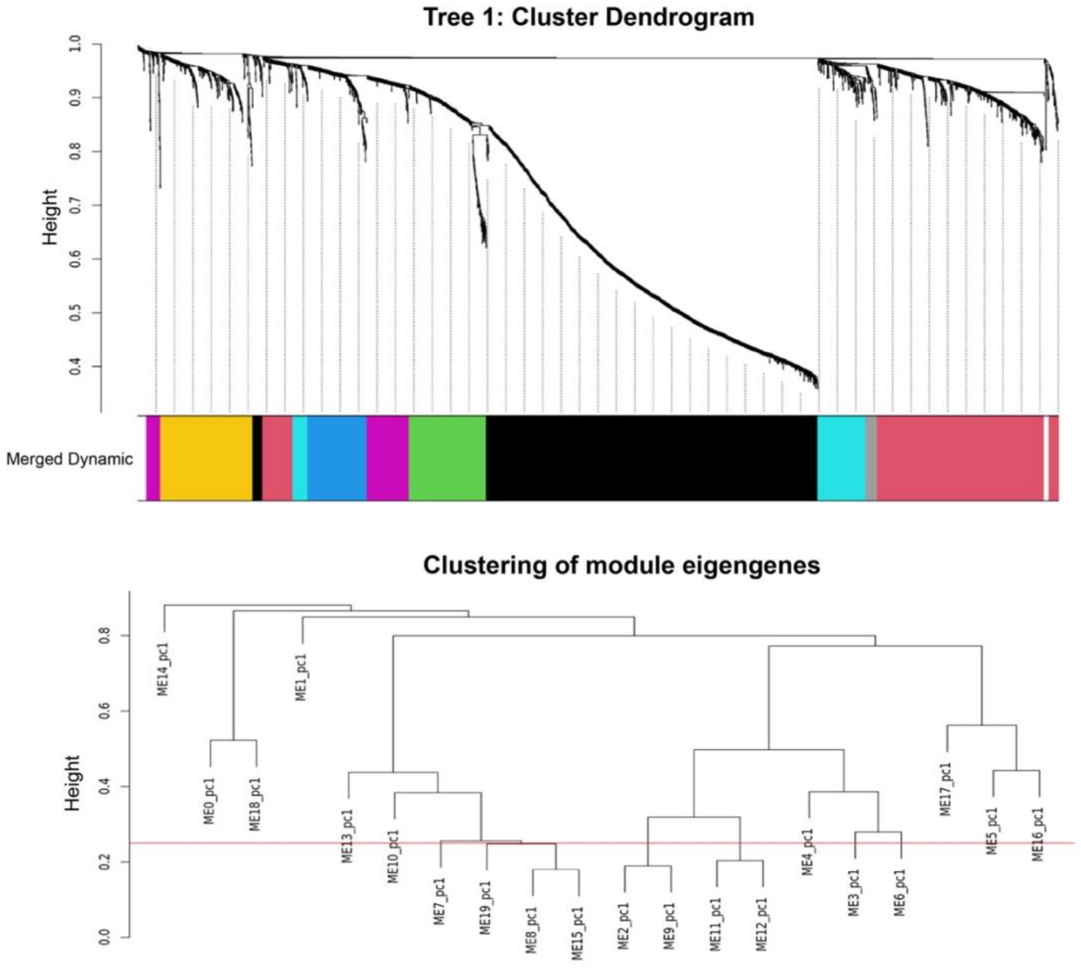


**Supplementary Fig. 15 Results of protein co-expression network construction.** Participants with missing protein data exceeding 50% were excluded. The remaining missing proteins were imputed using the k-nearest imputation, resulting in a dataset of 2,920 proteins from 46,850 participants. The minimum module size was set to 20, and the Spearman filter method was applied with a soft power of $\beta$=2, using an "unsigned" network approach in clustering. **A.** Hierarchical cluster tree of 2,920 proteins measured at baseline. The band displays the separation of proteins into 13 modules using Netboost clustering. **B.** Hierarchical cluster tree of 13 modules with a cut-off height of 0.25.

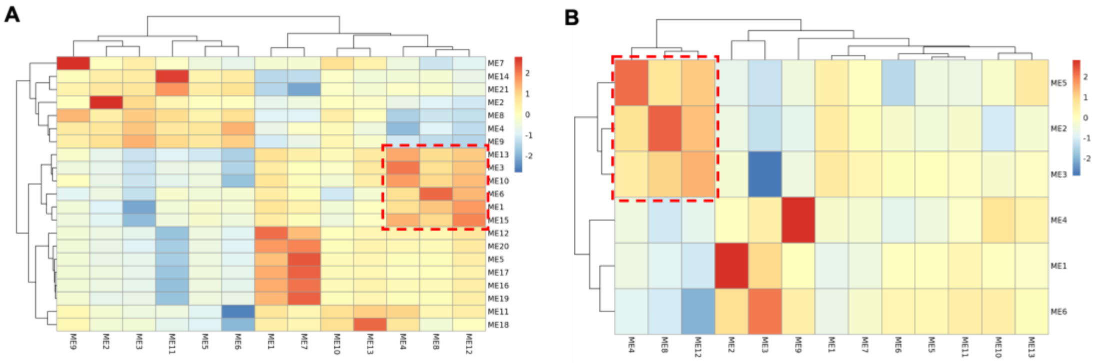


**Supplementary Fig. 16 Comparison between protein co-expression networks constructed using different parameters. A.** Heat map showing the association between module eigengenes identified by a soft power of β=2 (row) and β=3 (column). **B.** Heat map showing the association between module eigengenes identified by a soft power of β=2 (row) and β=null (column). An unsupervised hierarchical cluster analysis was used to group modules based on their associations.

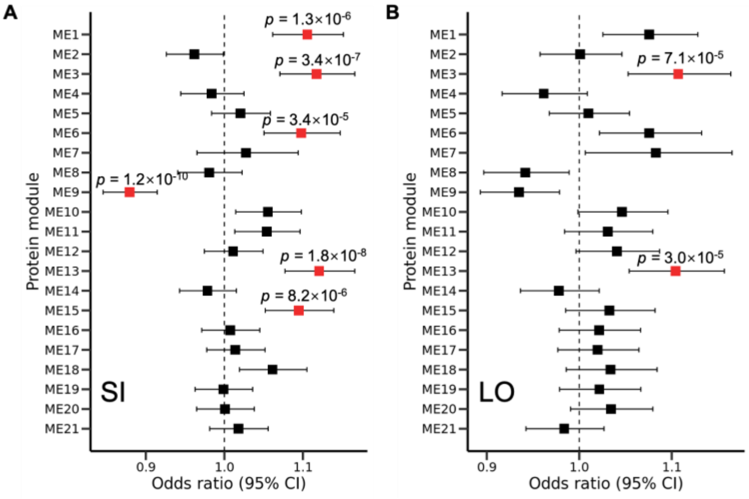


**C** **Module 1: 319 proteins**

| Term ID | Term name | FDR *q* |
|---|---|---|
| KEGG:04610 | Complement and coagulation cascades | 1.75E-12 |
| WP:WP558 | Complement and coagulation cascades | 1.22E-09 |
| REAC:R-HSA-166658 | Complement cascade | 1.68E-08 |
| REAC:R-HSA-977606 | Regulation of Complement cascade | 2.68E-08 |
| GO:0006956 | complement activation | 5.74E-06 |
| WP:WP2806 | Complement system | 7.04E-06 |
| REAC:R-HSA-140877 | Formation of Fibrin Clot (Clotting Cascade) | 1.14E-03 |
| WP:WP272 | Blood clotting cascade | 1.91E-03 |
| GO:0006807 | nitrogen compound metabolic process | 2.33E-03 |
| KEGG:05150 | Staphylococcus aureus infection | 8.86E-03 |
| GO:0006958 | complement activation, classical pathway | 1.23E-02 |
| GO:0004252 | serine-type endopeptidase activity | 2.21E-02 |
| GO:0048020 | CCR chemokine receptor binding | 2.21E-02 |
| GO:0017171 | serine hydrolase activity | 3.46E-02 |

**D** **Module 3: 375 proteins**

| Term ID | Term name | FDR *q* |
|---|---|---|
| GO:0060089 | molecular transducer activity | 8.86E-14 |
| GO:0038023 | signaling receptor activity | 8.86E-14 |
| GO:0004888 | transmembrane signaling receptor activity | 4.22E-10 |
| GO:0007155 | cell adhesion | 5.98E-08 |
| GO:0051241 | negative regulation of multicellular organismal process | 6.90E-07 |
| GO:0007166 | cell surface receptor signaling pathway | 6.90E-07 |
| KEGG:04060 | Cytokine-cytokine receptor interaction | 2.66E-06 |
| REAC:R-HSA-198933 | Immunoregulatory interactions between a Lymphoid and a non-Lymphoid cell | 4.55E-06 |
| WP:WP5078 | T cell modulation in pancreatic cancer | 3.81E-03 |
| REAC:R-HSA-168256 | Immune System | 1.21E-02 |
| REAC:R-HSA-5669034 | TNFs bind their physiological receptors | 1.21E-02 |
| KEGG:04380 | Osteoclast differentiation | 2.47E-02 |

**E** **Module 6: 81 proteins**

| Term ID | Term name | FDR *q* |
|---|---|---|
| GO:0006082 | organic acid metabolic process | 8.91E-09 |
| REAC:R-HSA-1430728 | Metabolism | 1.23E-08 |
| GO:0043436 | oxoacid metabolic process | 1.51E-08 |
| GO:0019752 | carboxylic acid metabolic process | 1.51E-08 |
| REAC:R-HSA-211859 | Biological oxidations | 6.99E-07 |
| KEGG:01100 | Metabolic pathways | 1.04E-05 |
| KEGG:00980 | Metabolism of xenobiotics by cytochrome P450 | 2.07E-05 |
| REAC:R-HSA-9748784 | Drug ADME | 1.70E-04 |
| GO:0003824 | catalytic activity | 2.19E-03 |
| KEGG:00982 | Drug metabolism - cytochrome P450 | 2.79E-03 |
| GO:0016614 | oxidoreductase activity, acting on CH-OH group of donors | 4.14E-03 |
| GO:0106435 | carboxylesterase activity | 8.38E-03 |
| WP:WP2884 | NRF2 pathway | 2.70E-02 |
| WP:WP136 | Phase I biotransformations non P450 | 2.70E-02 |
| WP:WP100 | Glutathione metabolism | 4.35E-02 |

**F** **Module 13: 42 proteins**

| Term ID | Term name | FDR *q* |
|---|---|---|
| GO:0008289 | lipid binding | 3.00E-03 |
| GO:0005504 | fatty acid binding | 9.04E-03 |
| GO:0033293 | monocarboxylic acid binding | 1.58E-02 |
| REAC:R-HSA-8979227 | Triglyceride metabolism | 3.69E-02 |
| REAC:R-HSA-163560 | Triglyceride catabolism | 3.69E-02 |
| WP:WP5061 | Fatty acid transporters | 4.56E-02 |

**G** **Module 15: 38 proteins**

| Term ID | Term name | FDR *q* |
|---|---|---|
| GO:0008289 | Neutrophil degranulation | 3.91E-07 |
| GO:0005504 | Innate Immune System | 1.50E-05 |
| GO:0033293 | Immune System | 1.49E-03 |
| REAC:R-HSA-8979227 | Antimicrobial peptides | 8.80E-03 |
| REAC:R-HSA-163560 | Interleukin-4 and Interleukin-13 signaling | 2.75E-02 |

**Supplementary Fig. 17 Association of protein co-expression network, constructed by soft power of β=3, with social isolation and loneliness (N = 35,475). A.** Forest plot showing the associations between protein modules and social isolation. Two-sided *P*-values were derived from logistic regression models adjusted for the same covariates as in the PWAS. Dots represent ORs, and error bars indicate 95% CIs. Red markers denote significance after Bonferroni correction ($P < 0.05/42 = 0.001$). **B.** Forest plot showing the associations between protein modules and loneliness. Enrichment analysis of proteins from modules 1 (**C**), 3 (**D**), 6 (**E**), 13 (**F**), and 15 (**G**). Top three significantly enriched pathways from each database are displayed. *P*-values are FDR-corrected.

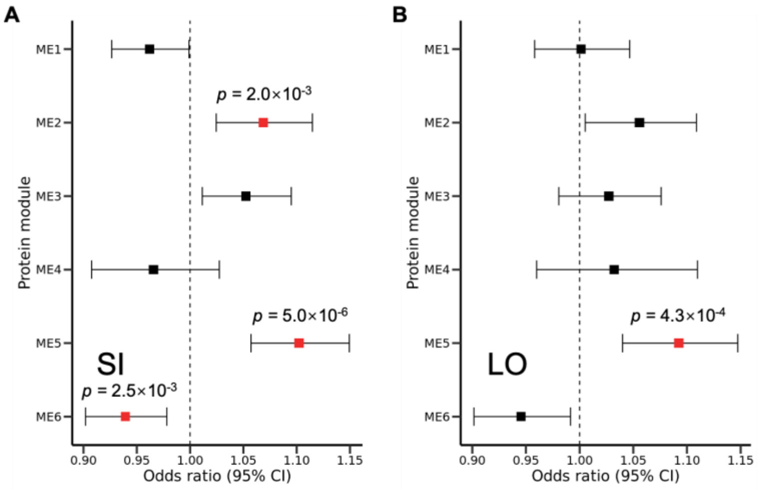


**C Module 2: 39 proteins**

| Term ID | Term name | FDR *q* |
|---|---|---|
| GO:0043436 | oxoacid metabolic process | 5.23E-14 |
| GO:0019752 | carboxylic acid metabolic process | 5.23E-14 |
| GO:0006082 | organic acid metabolic process | 5.23E-14 |
| REAC:R-HSA-1430728 | Metabolism | 3.56E-11 |
| KEGG:01100 | Metabolic pathways | 1.03E-08 |
| KEGG:00980 | Metabolism of xenobiotics by cytochrome P450 | 9.62E-08 |
| REAC:R-HSA-211859 | Biological oxidations | 1.05E-06 |
| REAC:R-HSA-71291 | Metabolism of amino acids and derivatives | 1.05E-06 |
| GO:0003824 | catalytic activity | 1.48E-06 |
| GO:0019842 | vitamin binding | 1.48E-06 |
| GO:0016614 | oxidoreductase activity, acting on CH-OH group of donors | 1.48E-06 |
| KEGG:00982 | Drug metabolism - cytochrome P450 | 6.27E-05 |
| WP:WP3925 | Amino acid metabolism | 1.43E-03 |
| WP:WP106 | Alanine and aspartate metabolism | 2.14E-03 |
| WP:WP2884 | NRF2 pathway | 2.29E-03 |

**D Module 5: 106 proteins**

| Term ID | Term name | FDR *q* |
|---|---|---|
| GO:0060089 | molecular transducer activity | 6.91E-08 |
| GO:0005031 | tumor necrosis factor receptor activity | 6.91E-08 |
| GO:0005035 | death receptor activity | 6.91E-08 |
| KEGG:04060 | Cytokine-cytokine receptor interaction | 7.71E-06 |
| REAC:R-HSA-5668541 | TNFR2 non-canonical NF-kB pathway | 3.10E-04 |
| REAC:R-HSA-5669034 | TNFs bind their physiological receptors | 7.44E-04 |
| GO:0007166 | cell surface receptor signaling pathway | 6.69E-03 |
| GO:0051241 | negative regulation of multicellular organismal process | 6.69E-03 |
| GO:0050896 | response to stimulus | 6.69E-03 |
| REAC:R-HSA-381426 | Regulation of Insulin-like Growth Factor (IGF) transport and uptake by Insulin-like Growth Factor Binding Proteins (IGFBPs) | 2.47E-02 |

**E Module 6: 34 proteins**

| Term ID | Term name | FDR *q* |
|---|---|---|
| REAC:R-HSA-110362 | POLB-Dependent Long Patch Base Excision Repair | 1.91E-04 |
| REAC:R-HSA-5685939 | HDR through MMEJ (alt-NHEJ) | 1.91E-04 |
| GO:0003676 | nucleic acid binding | 9.48E-04 |
| GO:0003684 | damaged DNA binding | 9.48E-04 |
| WP:WP4752 | Base excision repair | 1.20E-03 |
| WP:WP4946 | DNA repair pathways full network | 1.39E-03 |
| REAC:R-HSA-73933 | Resolution of Abasic Sites (AP sites) | 1.86E-03 |
| KEGG:03410 | Base excision repair | 2.57E-03 |
| GO:0097159 | organic cyclic compound binding | 3.16E-03 |
| WP:WP3644 | NAD metabolism | 3.26E-03 |
| KEGG:03040 | Spliceosome | 1.26E-02 |
| GO:0032200 | telomere organization | 2.77E-02 |
| GO:0051053 | negative regulation of DNA metabolic process | 2.77E-02 |
| GO:0006284 | base-excision repair | 2.77E-02 |

**Supplementary Fig. 18 Association of protein co-expression network, constructed by soft power of β=null, with social isolation and loneliness (N = 35,475). A.** Forest plot showing the associations between protein modules and social isolation. Two-sided *P*-values were derived from logistic regression models adjusted for the same covariates as in the PWAS. Dots represent ORs, and error bars indicate 95% CIs. Red markers denote significance after Bonferroni correction ($P < 0.05/12 = 0.004$). **B.** Forest plot showing the associations between protein modules and loneliness. Enrichment analysis of proteins from modules 2 (**C**), 5 (**D**), and 6 (**E**). Top three significantly enriched pathways from each database are displayed. *P*-values are FDR-corrected.

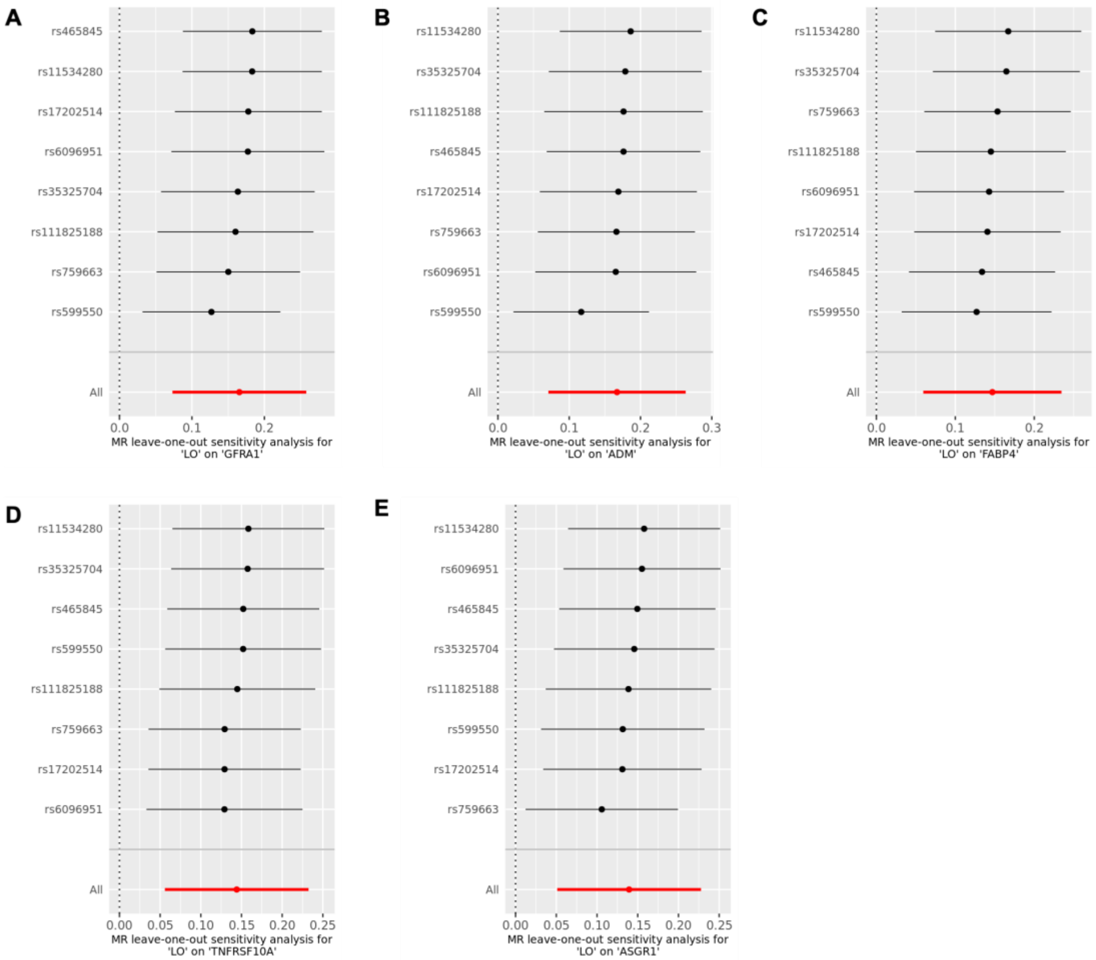


**Supplementary Fig. 19 Results of leave-one-out sensitivity analysis for MR using IVW method.** The GWAS sample sizes were 297,396 for social isolation, 288,696 for loneliness, 35,327 for GFRA1, 35,385 for ADM, 35,544 for FABP4, 34,842 for TNFRSF10A, and 34,842 for ASGR1. **A.** Leave-one-out IVW analyses of the MR between loneliness and GFRA1. Dots represent MR effect size, and error bars represent 95% CIs. The y-axis displays the excluded genetic variant per MR estimate. **B.** Leave-one-out IVW analyses of the MR between loneliness and ADM. **C.** Leave-one-out IVW analyses of the MR between loneliness and FABP4. **D.** Leave-one-out IVW analyses of the MR between loneliness and TNFRSF10A. **E.** Leave-one-out IVW analyses of the MR between loneliness and ASGR1.
