# Supplementary material for: Plasma proteomic signatures of social isolation and loneliness associated with morbidity and mortality
Source: Nat Hum Behav. 2025 Jan 3;9(3):569–83. doi: 10.1038/s41562-024-02078-1 (PMC11936835; doi:10.1038/s41562-024-02078-1)
Supplement: Supplementary file 2 — Reporting Summary [file 41562_2024_2078_MOESM2_ESM.pdf]

nature portfolio

Corresponding author(s): Jianfeng Feng
Wei Cheng
Barbara Sahakian

Last updated by author(s): Oct 23, 2024

Reporting Summary

Nature Portfolio wishes to improve the reproducibility of the work that we publish. This form provides structure for consistency and transparency in reporting. For further information on Nature Portfolio policies, see our Editorial Policies and the Editorial Policy Checklist.

Statistics

For all statistical analyses, confirm that the following items are present in the figure legend, table legend, main text, or Methods section.

n/a	Confirmed	
☐	☒	The exact sample size (n) for each experimental group/condition, given as a discrete number and unit of measurement
☐	☒	A statement on whether measurements were taken from distinct samples or whether the same sample was measured repeatedly
☐	☒	The statistical test(s) used AND whether they are one- or two-sided<br>*Only common tests should be described solely by name; describe more complex techniques in the Methods section.*
☐	☒	A description of all covariates tested
☐	☒	A description of any assumptions or corrections, such as tests of normality and adjustment for multiple comparisons
☐	☒	A full description of the statistical parameters including central tendency (e.g. means) or other basic estimates (e.g. regression coefficient) AND variation (e.g. standard deviation) or associated estimates of uncertainty (e.g. confidence intervals)
☐	☒	For null hypothesis testing, the test statistic (e.g. F, t, r) with confidence intervals, effect sizes, degrees of freedom and P value noted<br>*Give P values as exact values whenever suitable.*
☐	☒	For Bayesian analysis, information on the choice of priors and Markov chain Monte Carlo settings
☒	☐	For hierarchical and complex designs, identification of the appropriate level for tests and full reporting of outcomes
☐	☒	Estimates of effect sizes (e.g. Cohen's d, Pearson's r), indicating how they were calculated

Our web collection on statistics for biologists contains articles on many of the points above.

Software and code

Policy information about availability of computer code

Data collection	No software was used.
Data analysis	R version 4.2.0 was primarily used for the analyses in this study. Related R packages include: Protein co-expression analysis was performed using 'Netboost' (v2.4.1) (https://www.bioconductor.org/packages/release/bioc/html/netboost.html); Protein imputation for network analysis was performed using 'impute' (v1.70.0) (https://bioconductor.org/packages/release/bioc/html/impute.html); Functional enrichment was performed using 'gprofiler2' (v0.2.3) (https://cran.r-project.org/web/packages/gprofiler2/index.html); Protein GWASs were performed through GCTA (v1.94.1) (https://yanglab.westlake.edu.cn/software/gcta); Mendelian randomization analysis was performed using 'TwoSampleMR' (v0.5.7) (https://mrcieu.github.io/TwoSampleMR/), 'gsmr2' (v1.1.1) (https://yanglab.westlake.edu.cn/software/gsmr/), and 'cause' (v1.2.0) (https://github.com/jean997/cause); Colocalization was performed through 'coloc' (v5.2.3) (https://chr1swallace.github.io/coloc/); Preprocessing of NMR-metabolic data was performed using 'ukbnmr' (v2.2) (https://cran.r-project.org/web/packages/ukbnmr/index.html). Social isolation and loneliness GWASs were performed through PLINK 2.0 (https://www.cog-genomics.org/plink/2.0/). PPI was performed using STRING (v12.0) (https://string-db.org/cgi/input?sessionId=besj5Fp9GYc2&input_page_show_search=on). PPI network visualization was conducted by Cytoscape (v3.10.0) (https://cytoscape.org/). T1-weighted images were processed with Freesurfer (v6.0.0) (https://surfer.nmr.mgh.harvard.edu/). Custom scripts for the analyses have been made available through the following GitHub repository: https://github.com/chunshen617/Proteomics_loneliness.

For manuscripts utilizing custom algorithms or software that are central to the research but not yet described in published literature, software must be made available to editors and reviewers. We strongly encourage code deposition in a community repository (e.g. GitHub). See the Nature Portfolio guidelines for submitting code & software for further information.

Data

Policy information about availability of data

All manuscripts must include a data availability statement. This statement should provide the following information, where applicable:

- Accession codes, unique identifiers, or web links for publicly available datasets
- A description of any restrictions on data availability
- For clinical datasets or third party data, please ensure that the statement adheres to our policy

The data used in the present study are available from the UK Biobank (https://www.ukbiobank.ac.uk) with restrictions applied. Data were used under licence and are thus not publicly available. Details regarding registration for data access can be found at http://www.ukbiobank.ac.uk/register-apply/. The data used in this study were accessed from the UK Biobank under the application number 19542. GWAS summary statistics used can be found at the Figshare website (https://figshare.com/projects/GWAS_summary_data/224229) (ref. 111). European ancestry reference data from the 1000 Genomes Project can be found via https://github.com/getian107/PRScsx?tab=readme-ov-file.

Research involving human participants, their data, or biological material

Policy information about studies with human participants or human data. See also policy information about sex, gender (identity/presentation), and sexual orientation and race, ethnicity and racism.

Reporting on sex and gender	Both male and female subjects from the UK Biobank study were included. Sex (Data-Field 31) was obtained from the central registry at recruitment, but in some instances, it was updated by the participant. Consequently, this field may contain a blend of the sex recorded by the NHS for the participant and self-reported sex. Summary statistics on sex distributions were reported in Table 1. All statistical models were adjusted for sex, and sensitivity analyses including a sex interaction term and sex subgroup analysis were also conducted.
Reporting on race, ethnicity, or other socially relevant groupings	Table 1 presents the baseline characteristics of study participants, encompassing age, sex, ethnicity (white, mixed, Asian, black, and other), education level, and household income. Covariates were chosen based on literature and data availability. Two types of models, each with distinct covariates, were investigated in this study. The simple model comprised age, sex, site, technical factors, and the first 20 genetic PCs. Additionally, a fully adjusted model was explored, incorporating ethnicity, education level, household income, smoking, alcohol consumption, and BMI as covariates. The fully adjusted model was utilized for all primary analyses.
Population characteristics	Our primary study population included 42,062 participants (56.4±8.2 years and 52.3% female) from the UK Biobank, who had quality-controlled proteomic data and complete behavioral data including social isolation, loneliness, and all covariates. A flow chart of participant selection is shown in Supplementary Fig. 1. Among these, 3,905 (9.3%) reported as being socially isolated, and 2,689 (6.4%) felt lonely. Detailed demographic characteristics, stratified by social isolation and loneliness, are presented in Table 1. During a median (SD) follow-up of 13.7 (2.1) years (ended on November 30, 2022 or death), 2,695 participants developed CVD, 892 developed all-cause dementia, 1,703 developed T2D, 1,521 developed depression, 983 developed stroke, and 4,255 passed away.
Recruitment	The UK Biobank is a population-based cohort that involves over 500,000 individuals aged 40–69 years recruited from 22 centers across the UK between 2006 and 2010. Previous investigation have demonstrated a healthy volunteer bias in the UK Biobank.
Ethics oversight	All participants in the UK Biobank provided informed consent, and ethical approval was obtained from the National Information Governance Board for Health and Social Care and the North West Multi-Centre Research Ethics Committee (ref: 11/NW/0382).

Note that full information on the approval of the study protocol must also be provided in the manuscript.

Field-specific reporting

Please select the one below that is the best fit for your research. If you are not sure, read the appropriate sections before making your selection.

☒ Life sciences ☐ Behavioural & social sciences ☐ Ecological, evolutionary & environmental sciences

For a reference copy of the document with all sections, see nature.com/documents/nr-reporting-summary-flat.pdf

Life sciences study design

All studies must disclose on these points even when the disclosure is negative.

Sample size	No statistical methods were used to predetermine sample sizes and all currently available sample in the UK Biobank were used. Our primary study population included 42,062 participants (56.4±8.2 years and 52.3% female) from the UK Biobank, who had quality-controlled proteomic data and complete behavioral data including social isolation, loneliness, and all covariates.
Data exclusions	In survival analyses, we excluded prevalent cases, which were defined as diagnoses occurring within the first three years of follow-up or self-reported cases at baseline. This exclusion was implemented to reduce the risk of potential reverse-causation bias.<br>Genome-wide association studies (GWASs) on plasma protein abundance were conducted in Caucasian participants with qualified genotyping

data from the UK Biobank. To avoid over-fitting in two-sample MR, GWASs for social isolation (N=297,396) and loneliness (N=288,696) were performed using a distinct set of Caucasian participants, excluding those involved in the protein-related GWASs.

Replication

We replicated the primary analyses exclusively within the randomly selected subset (N=36,250). The proteins identified in this subset were consistent with those in all available samples, and the proteomic association patterns in these two populations were highly correlated (Supplementary Fig. 4). Additionally, to mitigate potential population stratification, we conducted PWAS specifically in Caucasians (N=35,697) and observed that the proteomic associative patterns were highly correlated with those found in the full sample (Supplementary Fig. 7). We also performed cross-validation by randomly splitting the UK Biobank samples 100 times. Our results showed that most of the significant proteins identified using the full sample retained significance in at least one of the two split samples, and proteomic associative patterns for social isolation and loneliness between the two split samples exhibited medium to large correlations (Supplementary Fig. 14).

Randomization

The primary analyses were controlled for age, sex, site, technical factors, ethnicity, education level, household income, smoking, alcohol consumption, BMI, and the first 20 genetic PCs. Additionally, two types of subgroup analyses were undertaken to examine potential sex (male vs female) and age (<60 vs >=60 years) differences.

Blinding

Blinding was not applicable to this study as this study is observational.

Reporting for specific materials, systems and methods

We require information from authors about some types of materials, experimental systems and methods used in many studies. Here, indicate whether each material, system or method listed is relevant to your study. If you are not sure if a list item applies to your research, read the appropriate section before selecting a response.

Materials & experimental systems

n/a	Involved in the study	
☐	☒	Antibodies
☒	☐	Eukaryotic cell lines
☒	☐	Palaeontology and archaeology
☒	☐	Animals and other organisms
☒	☐	Clinical data
☒	☐	Dual use research of concern
☒	☐	Plants

Methods

n/a	Involved in the study	
☒	☐	ChIP-seq
☒	☐	Flow cytometry
☐	☒	MRI-based neuroimaging

Antibodies

Antibodies used

The UK Biobank plasma samples were analysed using the Olink Explore 3072 proximity extension assay platform, which is based upon an in-solution binding of two polyclonal antibody pools to a target protein and subsequent hybridization and enrichment of two unique single-stranded DNA probes to create a double stranded barcode unique for the antigen. The platform consists of 2,941 immunoassays targeting 2,925 proteins. Each assay is based on a pair of polyclonal antibodies. The antibodies bind to different sites on the target protein and are labelled with single-stranded complementary oligonucleotides. If matching pairs of antibodies bind to the protein, the attached oligonucleotides hybridize, and are then measured using next-generation sequencing. Olink Explore 3072 consists of 8 panels of 384 assays analysed by next-generation sequencing.

Validation

Quality control protocol was implemented by the UK Biobank Pharma Proteomics Project, and was developed and approved by scientists across the thirteen participating biopharmaceutical companies, including Amgen, Alnylam, AstraZeneca, Biogen, Bristol Myers Squibb, Calico, Genentech, GlaxoSmithKline, Janssen (Johnson & Johnson), Novo Nordisk, Pfizer, Regeneron, and Takeda. More detailed information can be found in biobank.ndph.ox.ac.uk/ukb/ukb/docs/PPP_Phase_1_QC_dataset_companion_doc.pdf.

Plants

Seed stocks

Report on the source of all seed stocks or other plant material used. If applicable, state the seed stock centre and catalogue number. If plant specimens were collected from the field, describe the collection location, date and sampling procedures.

Novel plant genotypes

Describe the methods by which all novel plant genotypes were produced. This includes those generated by transgenic approaches, gene editing, chemical/radiation-based mutagenesis and hybridization. For transgenic lines, describe the transformation method, the number of independent lines analyzed and the generation upon which experiments were performed. For gene-edited lines, describe the editor used, the endogenous sequence targeted for editing, the targeting guide RNA sequence (if applicable) and how the editor was applied.

Authentication

Describe any authentication procedures for each seed stock used or novel genotype generated. Describe any experiments used to assess the effect of a mutation and, where applicable, how potential secondary effects (e.g. second site T-DNA insertions, mosiacism, off-target gene editing) were examined.

Magnetic resonance imaging

Experimental design

Design type	Structural MRI
Design specifications	The UK Biobank designed the imaging acquisition protocols including 6 modalities, covering structural, diffusion and functional imaging. The collection order is T1-weighted structural image, resting-state functional MRI, task functional MRI, T2-weighted FLAIR structural image, diffusion MRI and susceptibility-weighted imaging. The T1-weighted structural image was acquired using straight sagittal orientation for 5 minutes.
Behavioral performance measures	We used T1-weighted structural imaging, during which participants were not required to perform any tasks.

Acquisition

Imaging type(s)	T1-weighted structural imaging
Field strength	3T
Sequence & imaging parameters	The EPI-based acquisitions utilize simultaneous multi-slice (multiband) acceleration. UK Biobank uses pulse sequences and reconstruction code from the Center for Magnetic Resonance Research (CMRR), University of Minnesota https://www.cmrr.umn.edu/multiband. The resolution is 1x1x1 mm and the field of view is 208x256x256 matrix. Straight sagittal orientation is used. TR and TE are 2000ms and 2.01ms respectively. The flip angle is 8 deg. Detailed sequence and imaging parameters are openly available here: https://biobank.ndph.ox.ac.uk/showcase/showcase/docs/brain_mri.pdf.
Area of acquisition	Whole brain

Diffusion MRI ☐ Used ☒ Not used

Preprocessing

Preprocessing software	Imaging-derived phenotypes (IDPs) generated through an image-processing pipeline developed and run on behalf of the UK Biobank were used in this study (https://biobank.ctsu.ox.ac.uk/crystal/crystal/docs/brain_mri.pdf). T1-weighted images were processed using FreeSurfer. Where available, T2_FLAIR images were used in conjunction with T1-weighted images to achieve more accurate cortical modeling than with T1 alone. Surface atlases were employed to extract IDPs related to surface area, volume, and mean cortical thickness of standard atlas regions. The Qoala-T approach was used to assess the quality of FreeSurfer outputs, supported by manual checks for outputs near the quality threshold. Any FreeSurfer outputs failing quality control were excluded from the IDPs. Surface volumes extracted using the Desikan-Killiany atlas for 68 cortical regions and the aseg atlas for 16 subcortical regions were used in this study. The full processing pipeline is openly available here: https://doi.org/10.1016/j.neuroimage.2017.10.034 and https://www.nature.com/articles/nn.4393.
Normalization	Spatial normalization was performed whenever applicable as described in detail in Miller et al. Nature Neuroscience (2016) and Alfaro-Almagro et al. Neuroimage (2018).
Normalization template	1 mm resolution version of MNI152 template
Noise and artifact removal	Noise and artifact removal were described in detail in Miller et al. Nature Neuroscience (2016) and Alfaro-Almagro et al. Neuroimage (2018).
Volume censoring	No volume censoring performed on this data

Statistical modeling & inference

Model type and settings	Linear regression model was used to investigate the associations between proteins and IDPs. Covariates include age, sex, imaging collection site, batch, time gap between blood collection and protein measurement, ethnicity, education level, household income, smoking, alcohol consumption, BMI, the first 20 genetic PCs, ICV, and time gap between baseline and imaging collection.
Effect(s) tested	T-tests were used to derive the two-sided p value.

Specify type of analysis: ☐ Whole brain ☒ ROI-based ☐ Both

Anatomical location(s)	Surface volumes extracted using the Desikan-Killiany atlas for 68 cortical regions and the aseg atlas for 16 subcortical regions were used in this study.
Statistic type for inference (See Eklund et al. 2016)	No whole brain voxel-wised or cluster-based analyses involved in this study.

Correction	Both Bonferroni (P < 0.05/(84*5)) and FDR correction (q < 0.05) using the Benjamini-Hochberg procedure results are provided. For FDR correction, all tests are considered simultaneously.

Models & analysis

n/a	Involved in the study
☒	☐ Functional and/or effective connectivity
☒	☐ Graph analysis
☒	☐ Multivariate modeling or predictive analysis
